# Pakistan at the precipice: The looming threat of measles amidst the COVID-19 pandemic

**DOI:** 10.3389/fpubh.2022.1000906

**Published:** 2022-11-21

**Authors:** Samiuddin Tariq, Faizan Niaz, Yusra Afzal, Rabbia Tariq, Abdulqadir J. Nashwan, Irfan Ullah

**Affiliations:** ^1^Dow Medical College, Dow University of Health Sciences, Karachi, Pakistan; ^2^Hamad Medical Corporation, Doha, Qatar; ^3^Kabir Medical College, Gandhara University, Peshawar, Pakistan; ^4^Institute of Public Health and Social Science (IPH&SS), Khyber Medical University, Peshawar, Pakistan

**Keywords:** measles, COVID-19, pandemic, Pakistan, preparedness

## Abstract

The global focus on curbing the COVID-19 pandemic has reduced the overall immunization rates worldwide. This, coupled with increasing malnutrition and strained healthcare, has increased measles cases and mortality globally. Many countries are thus facing outbreaks, with Afghanistan having reported 372 deaths between January and September 2022. Therefore, Pakistan, a country in a similar economic state and the world's fourth highest reported measles cases in 2022, must take action. Moreover, the current flooded state of Pakistan and the subsequent mass movement of population, deterioration of health services, and worsened living conditions all contribute to put the country at a high risk of potentially devastating Measles outbreaks. With vaccination rates down by 42% since the start of the pandemic and the threat of an outbreak increasing daily, there is only so much time before the situation spirals out of control.

## Perspective

The measles virus belongs to the paramyxovirus family. As one of the most contagious diseases, measles spread *via* respiratory or aerosolized droplets and direct contact with oral and nasal secretions. Following infection, patients typically develop a maculopapular rash and a prodrome of cough, coryza, and conjunctivitis. The presence of blue-white patches on the oral mucosa is unique to measles, termed “Koplik spots,” which aid in its diagnosis. Measles is also associated with several more severe complications, such as diarrhea, pneumonia, encephalitis, and subacute sclerosing panencephalitis. The disease is mostly endemic in Africa and Asia, in countries such as Pakistan, Nigeria, and Afghanistan (1, 2).

Measles immunization is achieved *via* its incorporation in the Measles, Mumps, and Rubella (MMR) Vaccine. The MMR vaccine is administered twice, with a first dose at 12–15 months of age and a second at 4–6 years. This vaccine is a highly safe and effective intervention to prevent measles, with 95% of children that receive it at 12 months of age developing antibodies. This number becomes 99% in children that receive both doses. Before the commencement of the measles vaccination initiative in 1963, ~3–4 million people were infected annually in the United States, of which 400–500 cases were fatal; 1,000 developed encephalitis. Since then, the ubiquitous influence of the vaccine has led to a 99% reduction in occurrence. Between 2000 and 2018, measles vaccination saved an estimated 23.2 million lives; Deaths fell by 73% (1, 3).

The World Health Organization (WHO) recommends 95% vaccination coverage to achieve herd immunity (4). Vaccination campaigns raised global coverage of the measles-containing vaccine first dose (MCV1) from 72% in 2000 to 86% in 2019. However, the COVID-19 pandemic saw this figure decrease to 84% in 2020 as the number of children who were not administered the MCV1 showed the largest recorded yearly rise since 2000 (5). Overburdening of health systems led to the postponement of mass immunization programs. Fifty-seven of these campaigns, which had been set to begin at the start of the pandemic, remain postponed as of 1 April 2022, with 19 of them targeting measles. This has left 73 million children unvaccinated and at risk. The number of cases worldwide in the first 2 months of 2022 is up by 79% in comparison to the same period in 2021 (4).

The situation has worsened further in Pakistan, where the pre-pandemic coverage stood at just 73% for the first dose and 67% for the second (6). The province of Sindh reported an overall 51% drop in immunization visits during the COVID-19 lockdown compared to the previous 6 months (6). Redirection of resources from routine immunization, delays in vaccine supply, staff reluctance to work and parents to bring their children to vaccination centers out of fear of infection, and travel restrictions for outreach programs may have played a role in this decline (6). Despite lifting the lockdown and resumption of vaccination programs, only a 9% increase in routine vaccinations has been reported, bringing the overall decline to 42%. The majority of the government's efforts, however, remain focused on curbing the spread of COVID-19 (6).

**Figure 1:**
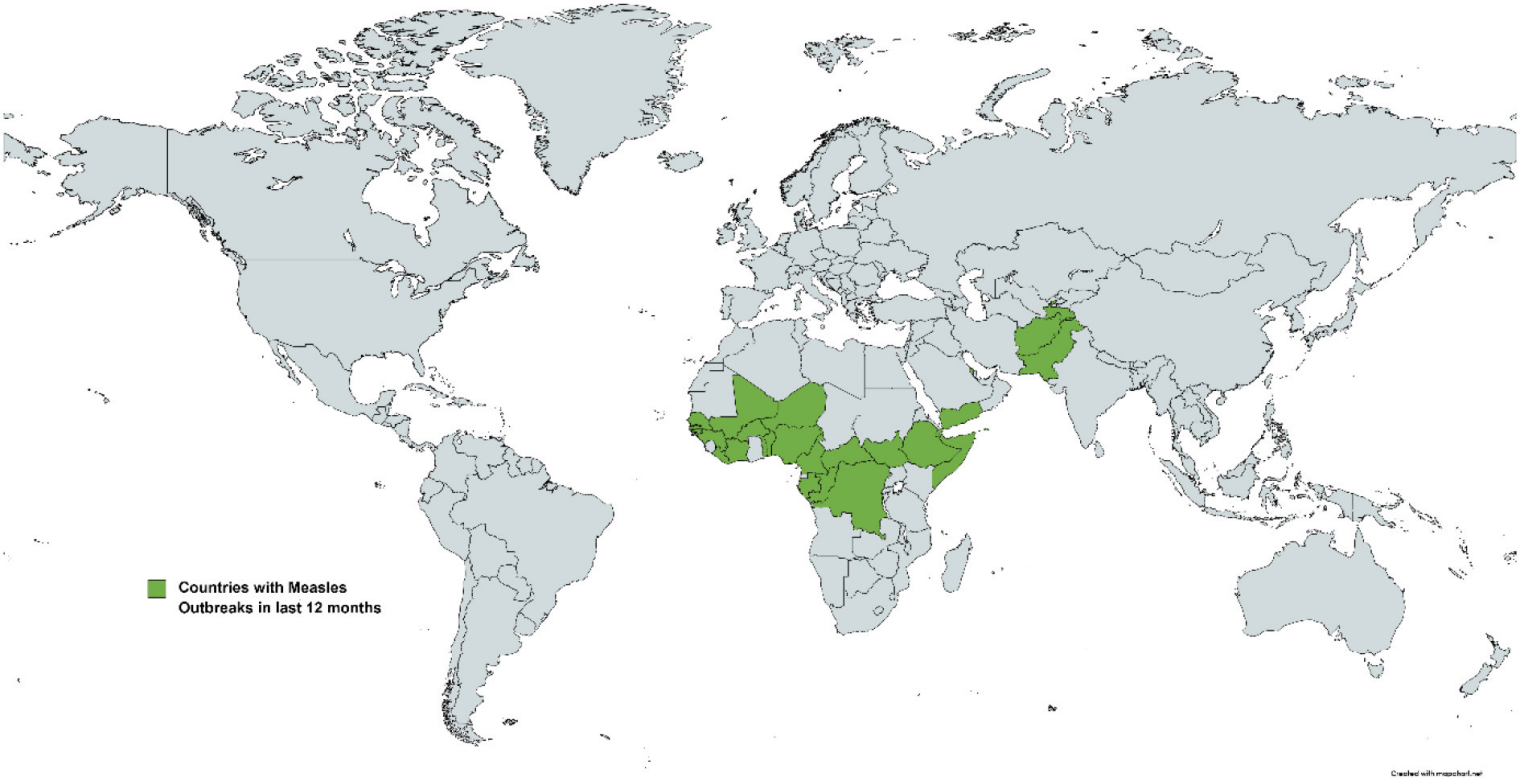
Countries with large measles outbreaks in the past 12 months.

In Afghanistan, between January and August 2022, there have been 67,855 suspected measles cases, causing 372 deaths (7). While there are only 3,514 confirmed cases, these are from 7,504 test samples, indicating a test positivity ratio of 46.8% (7). It is also important to remember that this may be an underestimation as many cases may go unreported. In contrast, only 156 confirmed deaths occurred in Afghanistan in 2021 (8). Nigeria is currently going through a similar trial, with 19,490 confirmed cases (9). As of September 2022, 26 countries have reported measles outbreaks in the past 12 months, depicted in Figure 1 (9). Among these countries, Liberia has reported the highest incidence rate, at 1,087 cases/1 million people (M) (9). Afghanistan stands at sixth among these, with 91 cases/M (9). Pakistan has reported 6,749 cases with an incidence rate of 29/M (9). Similar situations have been seen in other countries, such as Somalia, Ethiopia, and Yemen—all of which have suffered through disrupted immunization campaigns since the start of the pandemic (4).

Missed immunization, however, is not the only reason for this increase in measles cases and mortality. Research has shown that malnutrition correlates with increased viral infection severity (10). Particularly important is the intake of Vitamin A, which decreases mortality rates by 65% (10). Unfortunately, 190 million children under the age of five reportedly lack Vitamin A supplementation in low and middle-income countries (11). The effect of the pandemic, however, stretches beyond the community setting as health care has also been impacted (8, 10). It is worth noting that countries with recent outbreaks have strained healthcare systems. These overwhelmed healthcare systems, with understaffed workers, reduced drug availability, and crowded or poorly equipped hospitals, all contribute to raised mortality rates (12). Research has shown that mortality for non-COVID-19 patients has risen by 20% compared to before the pandemic (11). This number could be even higher for countries with impoverished healthcare systems, where measles is common.

In Pakistan's Sindh province, an estimated one out of every two children has missed routine immunization since the start of the COVID-19 pandemic (13). In 2021, 10,399 cases of measles were reported in Pakistan, whereas 2,747 were reported in 2020 (14). This is concerning in and of itself, but even more so when considering the possibility of missed diagnosis, which has become common in the wake of the pandemic's disrupted surveillance systems (4). Moreover, to make future measles outbreaks direr, 12% of Pakistan's population suffers from malnutrition—the fourth highest in the world (15). Even before the pandemic, nearly 54% of Pakistani children were malnourished—including for Vitamin A (16). Furthermore, in a study rating healthcare systems across the world in terms of access and quality, Pakistan ranked at 156th out of 194 (17).

Pakistan is currently flooded. Following record monsoon rains since June and unprecedented global warming, an estimated one-third of the country is submerged, affecting over 33 million people (18). Millions have lost their homes, and the subsequent large-scale population movements and congested living conditions find themselves at great risk of infectious diseases (18). This can be related to the 2010 flooding that Pakistan experienced. In this time, from 2010 to 2013, there was an approximate eightfold increase in estimated Measles cases, from 4,321 in 2010 to 40,923 in 2013 (19). The data thus suggests that the population of Pakistan is at a huge risk of Measles-related morbidity and mortality.

**Figure 2:**
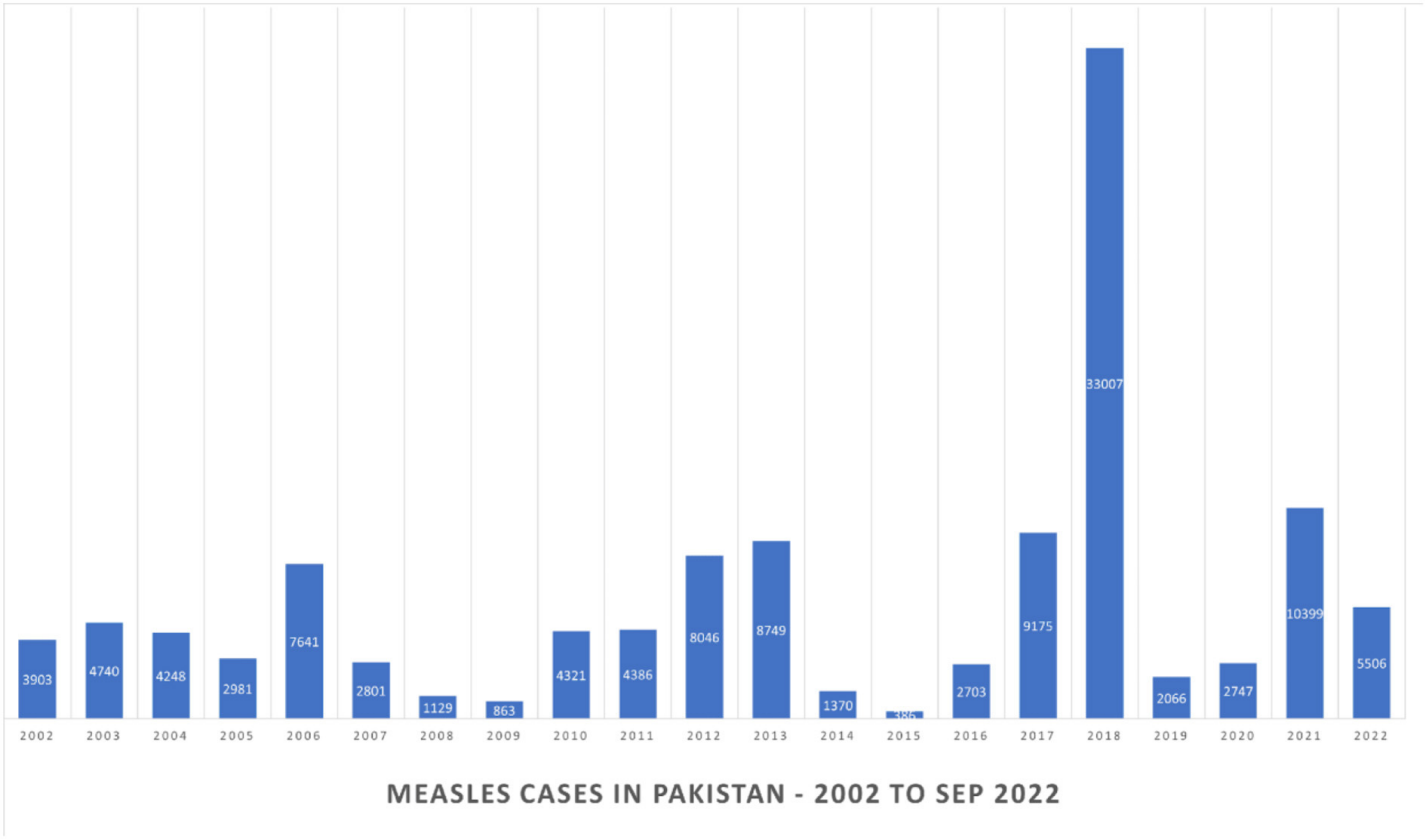
Reported measles cases in Pakistan—past 20 years.

Pakistan stands in fourth place alongside Nigeria, India, Liberia, and Ethiopia for the most reported measles cases between January 2022 till September 2022 (20). It is important to note that all of these countries, alongside Congo and Afghanistan, have recently suffered measles outbreaks as well (14). Since the start of the COVID-19 pandemic, Pakistan has seen an increase in the number of reported measles cases, from 2,066 in 2019 to 10,399 in 2021 (21). These are shown in Figure 2. While the government has made efforts to encourage immunization—including the world's largest campaign, in which 32 million children were immunized—a further commitment to this cause is necessary (22).

Measles is a vaccine-preventable disease. With due caution and planning and a refocused attitude toward raising immunization awareness by conducting vaccination programs, the virus's spread can be limited. In this post-COVID-19 era, with the population focusing on social distancing, door-to-door vaccination programs can be a valuable method to deal with lacking immunization numbers. The efficacy of door-to-door vaccination programs can be seen in its use in drought-struck Ethiopia, where 25 million children received the measles vaccine in a nationwide plan to curb the disease (23). Along with door-to-door vaccination, reducing the cost of the measles vaccine may help encourage immunization outside of state-wide vaccination programs and thus achieve the coverage needed to prevent future outbreaks (23). Furthermore, raised awareness of the protective effect of vitamin A supplementation can prove immensely useful in reducing the severity of measles infections (11).

Measles is among the top five causes of death in emergencies and disaster alongside diarrhea, acute respiratory infections, malnutrition, and malaria (24). It is thus important that the government of Pakistan employ the appropriate post-disaster management in order to reduce the risk of outbreaks (24). These include providing hygienic shelters with sufficient space and solid waste management, providing clean water and sanitation facilities, ensuring that disaster victims have access to clean, properly cooked, and preserved food (24). These victims must also receive proper vitamin A supplementation as well as a thorough immunization program with provision of essential clinical services to quickly screen, diagnose, treat, and prevent any diseases (24). With diligent screening and care, measles outbreaks can thus be prevented even in acute emergencies.

The COVID-19 pandemic has demonstrated the importance of caution and planning when dealing with outbreaks. While the virus may be sufficiently infectious and fatal, it is possible to plan for cases beforehand and diminish the threat in its infancy. In this aspect, as the shadow of another disease looms, Pakistan must focus on the task at hand and take action. If the current pandemic has taught us anything, you can never be too cautious.

## Data availability statement

The original contributions presented in the study are included in the article/supplementary material, further inquiries can be directed to the corresponding author.

## Author contributions

All authors equally contributed to the final write-up of the manuscript, draft review, editing, and approval of the final manuscript.

## Conflict of interest

Author AN was employed by Hamad Medical Corporation. The remaining authors declare that the research was conducted in the absence of any commercial or financial relationships that could be construed as a potential conflict of interest.

## Publisher's note

All claims expressed in this article are solely those of the authors and do not necessarily represent those of their affiliated organizations, or those of the publisher, the editors and the reviewers. Any product that may be evaluated in this article, or claim that may be made by its manufacturer, is not guaranteed or endorsed by the publisher.
